# Pubertal timing, depressive symptoms, and depression in adolescent males: a prospective cohort study

**DOI:** 10.1017/S0033291724003234

**Published:** 2025-02-04

**Authors:** Dana Tarif, Jon Heron, Abigail Fraser, Ahmed Elhakeem, Carol Joinson

**Affiliations:** Department of Population Health Sciences, University of Bristol, Bristol, UK

**Keywords:** Adolescence, ALSPAC, depression, puberty, pubertal timing

## Abstract

**Background:**

Early pubertal timing is associated with depressive symptoms in girls, but studies in boys are limited and have yielded conflicting results.

**Methods:**

N = 4,664 male participants from a UK birth cohort (Avon Longitudinal Study of Parents and Children – ALSPAC). Seven indicators of pubertal timing were measured repeatedly from 7 to 17 years (age at: peak height velocity, peak weight velocity, peak bone mineral content velocity, Tanner stage 3 pubic hair, Tanner stage 3 genitalia, axillary hair, and voice break), categorised into ‘early’, ‘on-time,’ and ‘late’ (mean ± 1 SD). Depressive symptoms (binary variable indicating higher versus lower levels) were assessed at 14 and 18 years, and depression (ICD-10 diagnosis) was assessed at 18 years. Multivariable logistic regression was used to examine associations between each indicator of pubertal timing and depressive symptoms/depression, adjusted for socioeconomic status (SES) and prepubertal body mass index (BMI).

**Results:**

Compared to males with normative pubertal development, the odds of depression at age 18 were higher in those with early age at peak height velocity (OR: 2.06; 95% CI 1.27–3.34), early age at peak weight velocity (OR: 2.10; 95% CI 1.16–3.79), and early age at Tanner genitalia stage 3 (OR: 1.81; 95% CI 1.01–3.26). There was no evidence for associations between pubertal timing and depressive symptoms at age 14 or 18.

**Conclusions:**

We found evidence that males with an earlier pubertal timing had increased odds of depression at age 18. Early maturing boys could be targeted for interventions aimed at preventing depression.

## Introduction

Adolescence is a critical developmental period associated with an increased risk of depressive symptoms and depression (Kwong, 2019; Thapar, Collishaw, Pine, & Thapar, 2012) Although girls experience a greater increase in the level of depressive symptoms during adolescence, an increase in depressive symptoms is also observed in boys during this period (Thapar et al., 2012). There is a robust association between early pubertal timing and depressive symptoms in adolescent girls (Galvao et al., 2014; Joinson, Heron, Araya, & Lewis, 2013; Ullsperger & Nikolas, 2017), but less research has been directed toward examining this association in boys. In children, the prevalence of depression is initially low, but rises notably around the onset of puberty, around age 12–14 (Kessler, Avenevoli, & Merikangas, 2001). Nearly 40% of individuals who experience depression have their first episode before age 20, underscoring adolescence as a critical period of vulnerability (Malhi & Mann, 2018). The relative lack of research on boys could be due to a focus on explaining the emergence of the unequal sex ratio in depressive symptoms/depression, which becomes more pronounced in girls during puberty (Thapar et al., 2012). Examining these associations in boys is essential as depressive symptoms that begin in adolescence often persist into adulthood, with implications for long-term mental health (Dekker et al., 2007).

Pubertal timing, defined as the relative onset of puberty in relation to same-age, same-sex peers, is often characterised in previous research as early, normative (on time), or late (Graber, 2013). The early timing hypothesis proposes that early pubertal timing is associated with adverse psychological outcomes (Brooks-Gunn & Warren, 1985). The off-timing hypothesis posits that both early and late pubertal timing are associated with adverse psychological outcomes (Caspi & Moffitt, 1991; Petersen, Crockett, Richards, & Boxer, 1988). The gendered deviation hypothesis is a sex-specific extension of the developmental deviance hypothesis (Brooks-Gunn & Warren, 1985; Sontag, Graber, & Clemans, 2011) which proposes that early puberty in girls and late puberty in boys are associated with an increased risk of negative psychological outcomes. As girls generally experience puberty earlier than boys (mean age = 11 and 13 years, respectively) (Patton & Viner, 2007), the early-maturing girls and late-maturing boys deviate most significantly from their opposite-sex peers.

Findings of previous studies examining associations between pubertal timing and depressive symptoms in males are inconsistent (Hamlat, McCormick, Young, & Hankin, 2020; Ullsperger & Nikolas, 2017). One longitudinal study found that both early and late pubertal timing (measured by self-reported axillary hair, facial hair, and voice break) were associated with depressed mood at age 12 (Kaltiala-Heino, Kosunen, & Rimpela, 2003; Natsuaki, Biehl, & Ge, 2009). Another longitudinal study found that early puberty is associated with depressive symptoms at age 15, utilising validated self-reported measurement scales; however, this study included only 81 male participants (Rudolph, Troop-Gordon, Lambert, & Natsuaki, 2014). Other longitudinal studies have reported an association between depressive symptoms and late pubertal timing (measured by self-reported axillary hair, facial hair, and voice break) at age 16 (Hoyt, Niu, Pachucki, & Chaku, 2020), or no association between pubertal timing and depressive symptoms at age 15 (Crockett, Carlo, Wolff, & Hope, 2013). A recent meta-analysis concluded there was no strong evidence that the effects of pubertal timing on males’ psychopathology persist into young adulthood (Ullsperger & Nikolas, 2017). However, most studies investigating pubertal timing and depressive symptoms/depression in males are limited to mid-adolescence, with few studies examining if any associations persist beyond puberty.

The comparative lack of studies examining puberty and depression in males could also be attributed to the difficulty in measuring the onset of puberty in males (Deardorff, Hoyt, Carter, & Shirtcliff, 2019). Unlike the widely used age at menarche (AAM) in females, there is no analogous event in males (Deardorff et al., 2019; Hayward, 2003). Age at spermarche/oigarche is difficult to measure due to its private nature (Deardorff et al., 2019) and age at voice break is hard to define as it occurs over several months. Self-reported measurement scales, such as the Tanner stages of development (Marshall & Tanner, 1970; Tanner, 1962) and the Pubertal Development Scale (PDS) (Petersen et al., 1988), can be subjective and less reliable (Dorn & Biro, 2011). However, age at peak height velocity (aPHV), the age when height increases most rapidly, provides an objective, noninvasive measure of pubertal timing that can be used in both sexes and correlates well with other indicators (Cole, Pan, & Butler, 2014; Roberts, Joinson, Gunnell, Fraser, & Mars, 2020).

The aim of this study is to investigate the associations between multiple prospectively collected measures of pubertal timing (age at: peak height velocity, peak weight velocity, peak bone mineral content (BMC) velocity, Tanner stage 3 pubic hair, Tanner stage 3 genitalia, axillary hair, and voice break), and depressive symptoms (14 and 18 years) and depression (18 years) in male participants in a UK cohort study.

## Methods

### Participants

The Avon Longitudinal Study of Parents and Children (ALSPAC) is a birth cohort that originally recruited pregnant women (*n =* 14,541) residing in Avon, UK with expected dates of delivery from 1st April 1991 to 31st December 1992. Of the initial pregnancies, there were 14,676 foetuses, 14,062 were live births, and 13,988 children who were alive at 1 year of age. When the oldest children were approximately 7 years of age, an attempt was made to increase the original sample by recruiting eligible individuals who did not join the study. This resulted in a total sample size of 15,454 pregnancies (15,658 foetuses, 14,901 alive at 1 year of age) when using data after the age of 7. Due to confidentiality reasons, data on 13 triplets/quads are not provided resulting in 15,645 cases (Boyd et al., 2013; Fraser et al., 2013; Northstone et al., 2019). Of these, 49.2% were male (assigned sex at birth), resulting in an initial sample of 7,684.

Please note that the study website contains details of all the data that is available through a fully searchable data dictionary and variable search tool (http://www.bristol.ac.uk/alspac/researchers/our-data/). Ethical approval for the study was obtained by ALSPAC Law and Ethics Committee and Local Research Ethics Committees. The authors assert that all procedures contributing to this work comply with the ethical standards of the relevant national and institutional committees on human experimentation and with the Helsinki Declaration of 1975, as revised in 2008.

### Measures

#### Depressive symptoms

Depressive symptoms were measured at 13.84 (SD = 0.21) and 17.84 (SD = 0.40) years old, hereafter referred to as 14 and 18 years, using the Short Moods and Feeling Questionnaire (SMFQ) (Angold et al., 1995), a widely used and validated measure of depressive symptoms in adolescents (Turner, Joinson, Peters, Wiles, & Lewis, 2014). The SMFQ is a 13-item questionnaire measuring the occurrence of depressive symptoms over the past 2 weeks. The total scores on the questionnaire range from 0 to 26, with higher scores indicating greater depressive symptoms. We used SMFQ scores of 11 and greater to indicate high levels of depressive symptoms because this threshold has previously been found to have good specificity for predicting depression (Kwong, 2019; Turner et al., 2014.

#### Depression

Depression was assessed at 17.82 (SD = 0.46) years old, hereafter referred to as 18 years, at research clinics using a computerised version of the Revised Clinical Interview Schedule (CIS-R) (Lewis, Pelosi, Araya, & Dunn, 1992), which is a widely used and validated self-report questionnaire that assesses depression in community samples (Clark et al., 2007; Turner et al., 2014). The CIS-R asks about a range of symptoms experienced over the past week that are used to generate a depression diagnosis according to the International Classification of Diseases (ICD-10) criteria (World Health Organization [WHO], 1992). The outcome in this study was any ICD-10 diagnosis of depression (mild, moderate, or severe) versus none.

#### Pubertal timing measures

Seven indicators of pubertal timing were derived from anthropometric measures and indicators of pubertal development collected from 7 to 17 years, these are: aPHV, age at peak weight velocity (aPWV), age at peak BMC velocity, age at Tanner pubic hair stage 3, age at Tanner genitalia stage 3, age at axillary hair and age at voice break. Data on height, weight, and BMC were collected in research clinics and self-reported Tanner stages, voice break and axillary hair were collected in questionnaires. Of the seven measures, three measures were derived using Superimposition by Translation and Rotation (SITAR) analysis (Cole, Donaldson, & Ben-Shlomo, 2010): aPHV, aPWV, and age at peak BMC velocity. The remaining four indicators were derived using nonlinear mixed effect models (described in detail elsewhere (Elhakeem et al., 2023)).

**Figure 1.**
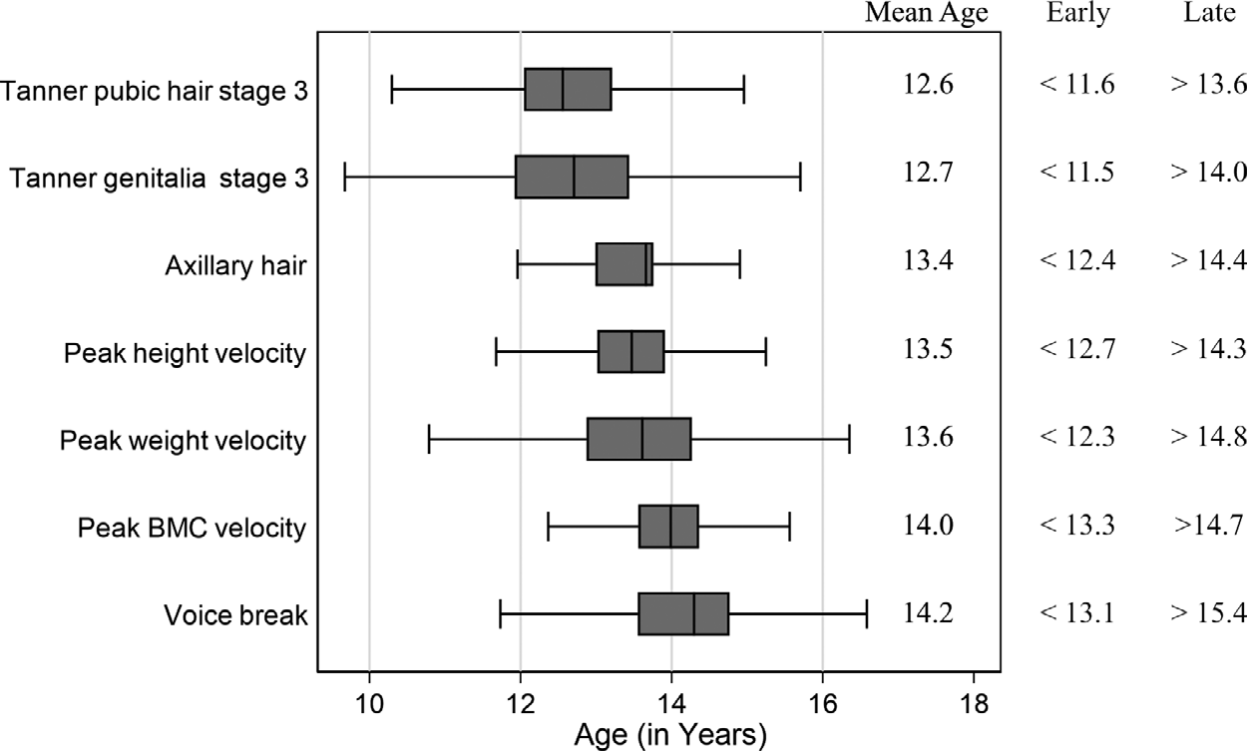
Timing of pubertal development in an imputed sample (N = 4664). Summary statistics including mean age and category cutoff points for each of the seven pubertal timing measures.

The seven measures of pubertal timing are expressed as age in years. We categorised each measure into ‘early’, ‘on-time’, and ‘late’, based on cutoff points defined as the mean ± 1 standard deviation (Figure 1), aligning with previous research (Roberts et al., 2020). Table 1 shows the distribution of each pubertal timing category in our sample. The use of categorical variables allowed us to examine potential nonlinear associations between pubertal timing and depressive outcomes. Using categorical variables allows for comparison with existing findings using this method and explores potential nonlinear associations between pubertal timing and depressive outcomes.

**Table 1.** Descriptive statistics in imputed sample (*N* = 4664)

		Mean (SE)	%	
			Early	Late
Age peak height velocity (in years)		13.5 (0.8)	18.4	15.3
Age peak weight velocity (in years)		13.6 (1.2)	16.1	17.0
Age peak BMC velocity (in years)		14.0 (0.7**)**	16.4	15.5
Age tanner pubic hair stage 3 (in years)		12.6 (1.0)	16.7	15.0
Age tanner genitalia stage 3 (in years)		12.7 (1.2)	16.1	15.2
Age voice break (in years)		14.2 (1.1)	13.5	13.7
Age axillary hair (in years)		13.4 (1.0)	20.0	10.5
BMI at 9		17.5 (2.7)		
		**% (SE)**		
Depressive symptoms at 14^a^		7.8 (0.5)		
Depressive symptoms at 18^a^		15.9 (0.9)		
Depression at 18		4.3 (0.5)		
Home ownership (renting/non-homeowner)		11.1 (0.5)		
Maternal education				
< O-levels (CSE/Vocational/None)		19.6 (0.6)		
O-Levels		41.2 (0.8)		
Major financial problems		25.4 (0.6)		
Social class		16.2 (0.6)		
Father absence				
< 5 years		8.2 (0.4)		
Between 5 and 10 years		14.3 (0.6)		

a *Depressive symptoms = SMFQ > =11*

#### Confounders

Socioeconomic status (SES) was measured by occupational class (manual versus nonmanual) during the antenatal period, home ownership status (renter versus owned/privately rented) at age 1.8 years, maternal educational attainment (Certificate of Secondary Education [CSE]/Vocational qualifications/none; O-Levels (equivalent to high school diploma); A-Levels; and above) at 5.1 years, major financial problems (experienced in first 5 years of child’s life versus. none) and father absence (before age 5, between age 5–10 or father present). Body mass index (BMI) measured at age 9 years was calculated based on height and weight measurements obtained from research clinics (90.2%) and self-report data when clinic data were missing (9.8%). Confounder selection was guided by empirical research on factors related to both pubertal timing and depression (Hoyt et al., 2020; Roberts et al., 2020).

### Data analysis

We assessed associations between the seven pubertal timing variables and depressive symptoms (at 14 and 18 years) and depression (at 18 years) separately using multivariable logistic regression analysis, adjusted for the confounders (unadjusted results are available in the Supplementary Material, Tables S1–3). All analyses were carried out using Stata 17 (StataCorp, 2023).

#### Missing data

4,664 participants had at least one puberty measure available and hence were eligible for inclusion in this study. Complete data on all puberty variables, all depression outcomes and all confounders were available for 986 participants (complete-case sample). Missing data were imputed using Multiple Imputation by Chained Equations (MICE) (Royston & White, 2011). Fifty datasets were imputed (25 iterations), with parameter estimated pooled according to Rubin’s rules (Rubin, 1987). The imputation model included all variables in the analyses and relevant auxiliary variables (Supplementary Material, Table S8). Separate imputations were conducted for each of the seven pubertal timing measures, while still including the other six measures as continuous auxiliary variables in each model.

## Results

Mean age at puberty varied from 12.6 years for Tanner’s pubic hair stage 3 to 14.2 years for voice break (Figure 1). The proportion or mean values for all puberty measures, confounders, and outcomes are shown in Table 1. Comparison of sample characteristics is shown in the Supplementary Material (Table S8).

### Presence of depressive symptoms

**Figure 2.**
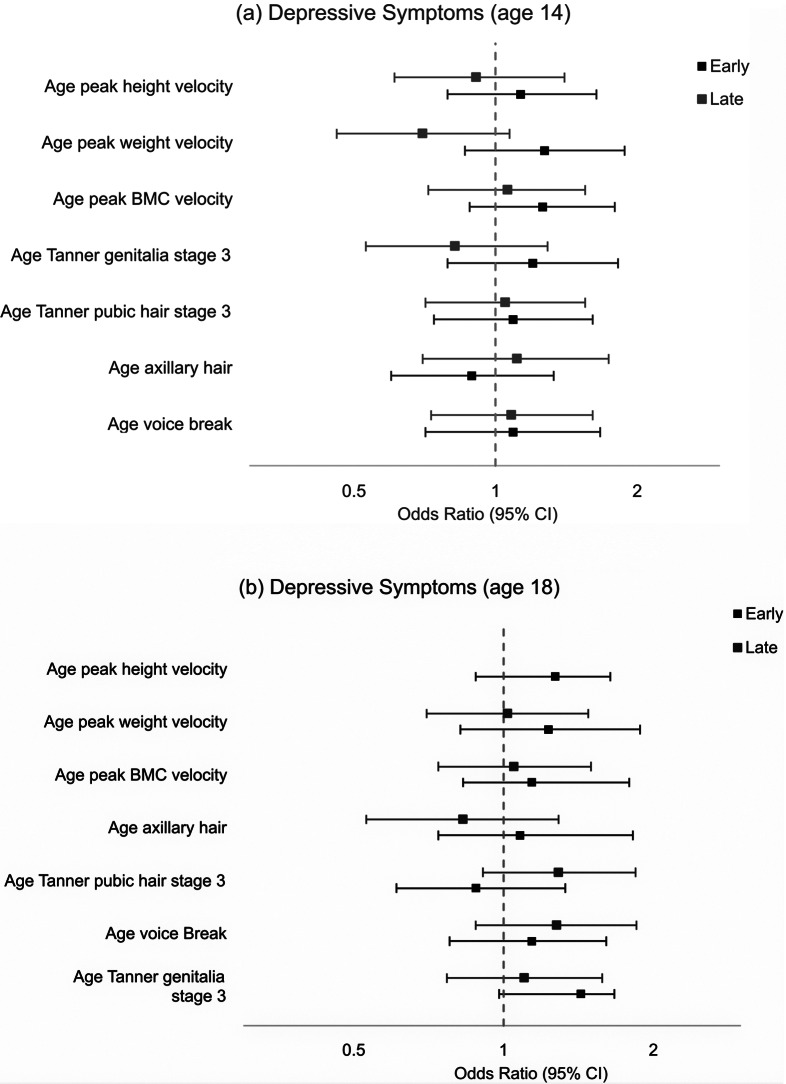
Association between pubertal timing variables and depressive symptoms at (a) 14 and (b) 18 years, adjusting for SES and BMI at 9, in an imputed sample (N = 4664). *Note:* Depressive symptoms = SMFQ > =11.

Figure 2 shows the adjusted associations between the seven pubertal timing indicators and depressive symptoms (SMFQ> = 11) at ages 14 and 18. There was no evidence that timing of puberty (earlier or later) was associated with depressive symptoms at age 14 and little evidence that pubertal timing was associated with depressive symptoms at 18 years. The exception was early, compared with on-time, age at voice break was weakly associated with increased odds of depressive symptoms at age 18 (AOR: 1.43; 95% CI 0.98, 2.09).

### Depression

**Figure 3.**
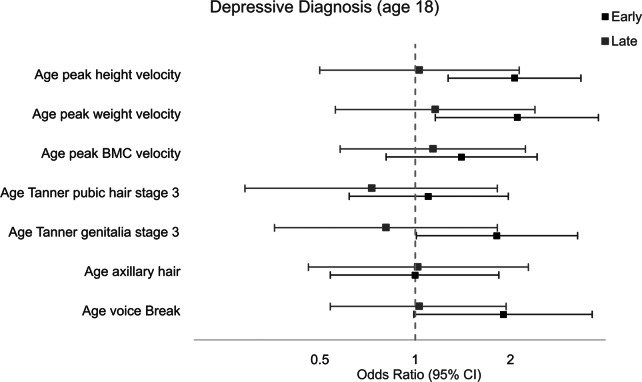
Association between pubertal timing variables and depressive symptoms at 18 years, adjusting for SES and BMI at 9, in an imputed sample (N = 4664).

Figure 3 shows the adjusted associations between the seven pubertal timing variables and depression at age 18. Compared to males with on-time pubertal development, those with early aPHV, aPWV, and early age at Tanner genitalia stage 3 had higher odds of depression (AOR: 2.06; 95% CI 1.27–3.34, AOR: 2.10; 95% CI 1.16–3.79, AOR: 1.81; 95% CI 1.01–3.26, respectively). There was weak evidence that an earlier, compared with normative, age at voice break was associated with increased odds of depression (AOR: 1.90; 95% CI 0.99–3.62). There was no evidence of an association between early pubertal timing and depression for age at peak BMC velocity, age at axillary hair, or age at Tanner pubic hair stage 3. There was no evidence for an increased risk of depression for males with late pubertal timing, compared to on-time peers.

## Discussion

### Summary of main findings

Our study found evidence of associations between earlier pubertal timing and an increased risk of depression at age 18 in males. The odds of depression were higher for males with earlier, compared with on-time, aPHV (< 12.7 years), aPWV (< 12.3 years), and Tanner genitalia stage 3 (<11.5 years). There was no evidence for an association between pubertal timing and depressive symptoms at age 14 and little evidence for an association at age 18, except for age at voice break.

### Comparison with earlier findings

Previous research into pubertal timing and depressive symptoms/depression in boys has yielded inconclusive results, potentially due to a lack of homogeneity of methodology and measures of pubertal timing (Hayward, 2003; Hoyt et al., 2020). aPHV is emerging as a reliable and objective measure of pubertal timing which can be used in both boys and girls, yet no studies to date have utilised this measure when examining associations with depressive symptoms during adolescence. The inconsistency between our findings and some previous studies may be due to differences in measurement, with previous research relying on self-reported measures of pubertal timing (Hamlat et al., 2020; Natsuaki et al., 2009). Few studies have examined the association between pubertal timing and depression beyond adolescence. We found evidence of an association between pubertal timing and depression at age 18, in contrast with previous research indicating no association (Graber, Seeley, Brooks-Gunn, & Lewinsohn, 2004). Our findings are in line with the early timing hypothesis (Brooks-Gunn & Warren, 1985), suggesting that early maturing males are at heightened risk for adverse outcomes. Given the limited investigation of the longevity of pubertal timing effects, further research should be conducted to examine whether this association persists into adulthood.

It is notable that we found little evidence of an association between an early pubertal timing and depressive symptoms at age 18, but evidence that early pubertal timing was associated with CIS-R depression at age 18. The inconsistency in the results at age 18 may be explained by the potential masking of symptoms when measuring depressive symptoms via the SMFQ compared to the ICD-10 diagnosis of depression captured by the CIS-R. Gender differences in depression symptom presentation are well-documented, with males reporting more irritability, disturbance in sleep, and suicidal thoughts, whereas females report more sadness, guilt, and worthlessness (Khesht-Masjedi, Shokrgozar, Abdollahi, Golshahi, & Sharif-Ghaziani, 2017). Although the SMFQ and the CIS-R have demonstrated good predictive validity in the ALSPAC cohort (Turner et al., 2014, there are three symptoms missing from the SMFQ that are captured by the CIS-R (sleep disturbance, changes in appetite and suicidal ideation). Although females are twice as likely to be diagnosed with depression, males are three times more likely to die by suicide (Bachmann, 2018). This discrepancy might contribute to underestimating depressive symptoms in males, potentially due to the masking of symptoms in line with masculine stereotypes (Branney & White, 2008; Martin, Neighbors, & Griffith, 2013).

Our study found only three out of seven pubertal timing variables were associated with depression at age 18. These measures, while representing individual proxies for pubertal timing, are measured with varying degrees of measurement accuracy, as discussed within the limitations. One explanation for the inconsistency across variables is that these measurement issues apply to some measures (e.g. Tanner stages of development or voice break) more than others (e.g. aPHV). A second explanation is that the association between pubertal timing and depression may stem from different psychosocial and hormonal mechanisms, captured by different pubertal indicators. The early development of visible markers of maturation such as aPHV, compared with invisible markers such as peak BMC velocity, may subject boys to more adult-like treatment due to a mature appearance. Additionally, distinct hormonal processes driving different secondary sexual characteristics, such as testosterone influencing genitalia development and growth hormone spurring the growth spurt, could offer possible mechanisms for the association between pubertal timing and depression (Chronister et al., 2021; Copeland, Worthman, Shanahan, Costello, & Angold, 2019).

### Strengths and limitations

A major strength of this study is the prospective cohort study design and the inclusion of multiple indicators of pubertal timing, some of which were derived from objective measures enabling more reliable and valid indicators than previous research relying on self-reported measures. Additionally, multiple measures across childhood and adolescence enhanced the accuracy of determining pubertal timing, compared to studies relying on retrospective recall (Beltz, 2018). We adjusted for empirically relevant confounders, enabled by the availability of repeated, longitudinal measures within the ALSPAC cohort. A further strength is the assessment of depression during mid-adolescence, where there is a high degree of interindividual variation in pubertal maturation, and at age 18, when most boys will have gone through the pubertal transition. Not all boys will have undergone key pubertal events at age 14 when depressive symptoms were measured, however, it is crucial to recognise that pubertal timing indicators represent a continuum of pubertal development rather than a discrete event (Hayward, 2003). By including measurements at age 14, we assessed the association between pubertal timing (whether early, on-time or late) and depressive symptoms during this pivotal period of mid-adolescence, even for those who experience these pubertal events after this time point.

Despite these strengths, challenges arise due to the subjective nature of measurements, the lack of salient markers in males, and the intimate nature of specific measures (Deardorff et al., 2019; Hayward, 2003). For instance, unlike menarche, the gradual occurrence of voice breaking in males is difficult to measure. Missing data is a common challenge faced in cohort studies. Loss to follow-up can lead to selection bias, particularly as excluded individuals tend to be more socioeconomically disadvantaged compared with the original cohort, and there is a higher prevalence of depression among individuals with a lower SES (Freeman et al., 2016). Restricting the analysis to the sample with complete data could cause bias, in addition to loss of statistical power due to a smaller sample size. We therefore used multiple imputations to address potential bias due to attribution and missing data and reported the results from the analysis of the imputed data as the main findings. Multiple imputation has been found to eliminate bias regardless of the proportion of missing data (Madley-Dowd, Hughes, Tilling, & Heron, 2019). The categorisation of continuous pubertal timing data may be considered a limitation, as this reduces statistical power and introduces arbitrary cut-off points (Mendle, Beltz, Carter, & Dorn, 2019). However, this approach allowed us to examine potential nonlinear associations, addressing ambiguities in previous research regarding whether early or late pubertal timing is associated with depressive outcomes. Previous research has similarly used this categorisation, aiding the practical interpretation of the results. Finally, the ALSPAC participants are predominantly of White ethnicity (95%), limiting the generalisability of findings to other ethnic groups.

### Implications and future directions

Understanding the relationship between pubertal timing and mental health in males has significance for clinical practice and future research directions. The variability in associations of different pubertal indicators with depression has implications for future cohort studies wishing to utilise objective measures of pubertal timing in boys. Further research exploring the underlying mechanisms driving this association is warranted to inform targeted interventions for at-risk individuals. Recognising the divergence in depression symptomology between sexes emphasises the necessity of investigating the emergence and experience of male depression, despite the lower prevalence compared to females across the lifespan. Future researchers may wish to refine measurement instruments to include male-specific manifestations of depression.

### Conclusion

This study contributes to our understanding of the association between pubertal timing and depression in males. The findings indicate that there is evidence for an association between an earlier pubertal timing in males and increased odds of depression at age 18 and that there are differential associations across different pubertal timing indicators. The lack of evidence of associations between pubertal timing and depressive symptoms in males may be due to differences in the symptomatology of depression in adolescent males compared with females. Future cohort studies wishing to examine psychopathology and pubertal development should consider using multiple, repeated measures of puberty such as aPHV. Further research is needed to determine whether these associations are causal and identify potential mechanisms involved.

## Supporting information

Tarif et al. supplementary material Tarif et al. supplementary material

## Supplementary material

The supplementary material for this article can be found at http://doi.org/10.1017/S0033291724003234.

## References

[r1] Angold, A., Costello, E. J., Messer, S. C., Pickles, A., Winder, F., & Silver, D. (1995). Development of a short questionnaire for use in epidemiological studies of depression in children and adolescents. International Journal of Methods in Psychiatric Research, 5(4), 237–249. Retrieved from <Go to ISI>://WOS:A1995TQ48000002

[r2] Bachmann, S. (2018). Epidemiology of suicide and the psychiatric perspective. The International Journal of Environmental Research and Public Health, 15(7). 10.3390/ijerph15071425 PMC6068947 29986446

[r3] Beltz, A. M. (2018). Gendered mechanisms underlie the relation between pubertal timing and adult depressive symptoms. Journal of Adolescent Health, 62(6), 722–728. 10.1016/j.jadohealth.2017.12.019 29784116

[r4] Boyd, A., Golding, J., Macleod, J., Lawlor, D. A., Fraser, A., Henderson, J., … Davey Smith, G. (2013). Cohort Profile: the ‘children of the 90s’--the index offspring of the Avon longitudinal study of parents and children. International Journal of Epidemiology, 42(1), 111–127. 10.1093/ije/dys064 22507743 PMC3600618

[r5] Branney, P., & White, A. (2008). Big boys don’t cry: Depression and men. Advances in Psychiatric Treatment, 14(4), 256–262.

[r6] Brooks-Gunn, J., & Warren, M. P. (1985). Measuring physical status and timing in early adolescence – a developmental perspective. Journal of Youth and Adolescence, 14(3), 163–189. 10.1007/Bf02090317 24301175

[r7] Caspi, A., & Moffitt, T. E. (1991). Individual differences are accentuated during periods of social change: the sample case of girls at puberty. Journal of Personality and Social Psychology, 61(1), 157–168. 10.1037/0022-3514.61.1.157 1890586

[r8] Chronister, B. N., Gonzalez, E., Lopez-Paredes, D., Suarez-Torres, J., Gahagan, S., Martinez, D., … Suarez-Lopez, J. R. (2021). Testosterone, estradiol, DHEA and cortisol in relation to anxiety and depression scores in adolescents. Journal of Affective Disorders, 294, 838–846. 10.1016/j.jad.2021.07.026 34375211 PMC8992006

[r9] Clark, C., Rodgers, B., Caldwell, T., Power, C., & Stansfeld, S. (2007). Childhood and adulthood psychological ill health as predictors of midlife affective and anxiety disorders – The 1958 British Birth Cohort. Archives of General Psychiatry, 64(6), 668–678. 10.1001/archpsyc.64.6.668 17548748

[r10] Cole, T. J., Donaldson, M. D., & Ben-Shlomo, Y. (2010). SITAR – a useful instrument for growth curve analysis. International Journal of Epidemiology, 39(6), 1558–1566. 10.1093/ije/dyq115 20647267 PMC2992626

[r11] Cole, T. J., Pan, H., & Butler, G. E. (2014). A mixed effects model to estimate timing and intensity of pubertal growth from height and secondary sexual characteristics. Annals of Human Biology, 41(1), 76–83. 10.3109/03014460.2013.856472 24313626 PMC3877946

[r12] Copeland, W. E., Worthman, C., Shanahan, L., Costello, E. J., & Angold, A. (2019). Early pubertal timing and testosterone associated with higher levels of adolescent depression in girls. Journal of the American Academy of Child & Adolescent Psychiatry, 58(12), 1197–1206. 10.1016/j.jaac.2019.02.007 30768421 PMC6693999

[r13] Crockett, L. J., Carlo, G., Wolff, J. M., & Hope, M. O. (2013). The role of pubertal timing and temperamental vulnerability in adolescents’ internalizing symptoms. Development and Psychopathology, 25(2), 377–389. 10.1017/S0954579412001125 23627951

[r14] Deardorff, J., Hoyt, L. T., Carter, R., & Shirtcliff, E. A. (2019). Next steps in puberty research: Broadening the lens toward understudied populations. Journal of Adolescent Research, 29(1), 133–154. 10.1111/jora.12402 PMC6827435 30869847

[r15] Dekker, M. C., Ferdinand, R. F., van Lang, N. D., Bongers, I. L., van der Ende, J., & Verhulst, F. C. (2007). Developmental trajectories of depressive symptoms from early childhood to late adolescence: Gender differences and adult outcome. Journal of Child Psychology and Psychiatry, 48(7), 657–666. 10.1111/j.1469-7610.2007.01742.x 17593146

[r16] Dorn, L. D., & Biro, F. M. (2011). Puberty and its measurement: A decade in review. Journal of Research on Adolescence, 21(1), 180–195. 10.1111/j.1532-7795.2010.00722.x

[r17] Elhakeem, A., Frysz, M., Goncalves Soares, A. L., Bell, J., Cole, T., Heron, J., … Timpson, N. J. (2023). Evaluation and comparison of nine growth-and development-based measures of pubertal timing. Communications Medicine, 2023.2006. 2012.23290796. 10.1038/s43856-024-00580-1 PMC11306255 39112679

[r18] Fraser, A., Macdonald-Wallis, C., Tilling, K., Boyd, A., Golding, J., Davey Smith, G., … Lawlor, D. A. (2013). Cohort profile: The avon longitudinal study of parents and children: ALSPAC mothers cohort. International Journal of Epidemiology, 42(1), 97–110. 10.1093/ije/dys066 22507742 PMC3600619

[r19] Freeman, A., Tyrovolas, S., Koyanagi, A., Chatterji, S., Leonardi, M., Ayuso-Mateos, J. L., … Haro, J. M. (2016). The role of socio-economic status in depression: Results from the COURAGE (aging survey in Europe). BMC Public Health, 16(1), 1098. 10.1186/s12889-016-3638-0 27760538 PMC5069819

[r20] Galvao, T. F., Silva, M. T., Zimmermann, I. R., Souza, K. M., Martins, S. S., & Pereira, M. G. (2014). Pubertal timing in girls and depression: A systematic review. Journal of Affective Disorders, 155, 13–19. 10.1016/j.jad.2013.10.034 24274962

[r21] Graber, J. A. (2013). Pubertal timing and the development of psychopathology in adolescence and beyond. Hormones and Behavior, 64(2), 262–269. 10.1016/j.yhbeh.2013.04.003 23998670

[r22] Graber, J. A., Seeley, J. R., Brooks-Gunn, J., & Lewinsohn, P. M. (2004). Is pubertal timing associated with psychopathology in young adulthood. Journal of the American Academy of Child & Adolescent Psychiatry, 43(6), 718–726. 10.1097/01.chi.0000120022.14101.11 15167088

[r23] Hamlat, E. J., McCormick, K. C., Young, J. F., & Hankin, B. L. (2020). Early pubertal timing predicts onset and recurrence of depressive episodes in boys and girls. Journal of Child Psychology and Psychiatry, 61(11), 1266–1274. 10.1111/jcpp.13198 32017111 PMC7396277

[r24] Hayward, C. (2003). Methodological concerns in puberty-related research. In C. Hayward (Ed.), Gender differences at puberty (pp. 1–14). Cambridge: Cambridge University Press.

[r25] Hoyt, L. T., Niu, L., Pachucki, M. C., & Chaku, N. (2020). Timing of puberty in boys and girls: Implications for population health. SSM Population Health, 10, 100549. 10.1016/j.ssmph.2020.100549 32099893 PMC7030995

[r26] Joinson, C., Heron, J., Araya, R., & Lewis, G. (2013). Early menarche and depressive symptoms from adolescence to young adulthood in a UK cohort. Journal of the American Academy of Child & Adolescent Psychiatry, 52(6), 591–598 e592. 10.1016/j.jaac.2013.03.018 23702448

[r27] Kaltiala-Heino, R., Kosunen, E., & Rimpela, M. (2003). Pubertal timing, sexual behaviour and self-reported depression in middle adolescence. Journal of Adolescence, 26(5), 531–545. 10.1016/S0140-1971(03)00053-8 12972267

[r28] Kessler, R. C., Avenevoli, S., & Merikangas, K. R. (2001). Mood disorders in children and adolescents: An epidemiologic perspective. Biological Psychiatry, 49(12), 1002–1014. Doi 10.1016/S0006-3223(01)01129-5 11430842

[r29] Khesht-Masjedi, M. F., Shokrgozar, S., Abdollahi, E., Golshahi, M., & Sharif-Ghaziani, Z. (2017). Comparing depressive symptoms in teenage boys and girls. Journal of Family Medicine and Primary Care, 6(4), 775–779. 10.4103/jfmpc.jfmpc_129_17 PMC5848397 29564262

[r30] Kwong, A. S. F. (2019). Examining the longitudinal nature of depressive symptoms in the avon longitudinal study of parents and children (ALSPAC). Wellcome Open Research, 4, 126. 10.12688/wellcomeopenres.15395.2 31595229 PMC6764237

[r31] Kwong, A. S. F., Manley, D., Timpson, N. J., Pearson, R. M., Heron, J., Sallis, H., … Leckie, G. (2019). Identifying critical points of trajectories of depressive symptoms from childhood to young adulthood. Journal of Youth and Adolescence, 48(4), 815–827. 10.1007/s10964-018-0976-5 30671716 PMC6441403

[r32] Lewis, G., Pelosi, A. J., Araya, R., & Dunn, G. (1992). Measuring psychiatric disorder in the community: A standardized assessment for use by lay interviewers. Psychological Medicine, 22(2), 465–486. 10.1017/s0033291700030415 1615114

[r33] Madley-Dowd, P., Hughes, R., Tilling, K., & Heron, J. (2019). The proportion of missing data should not be used to guide decisions on multiple imputation. Journal of Clinical Epidemiology, 110, 63–73. 10.1016/j.jclinepi.2019.02.016 30878639 PMC6547017

[r34] Malhi, G. S., & Mann, J. J. (2018). Depression. Lancet, 392(10161), 2299–2312. 10.1016/S0140-6736(18)31948-2 30396512

[r35] Marshall, W. A., & Tanner, J. M. (1970). Variations in the pattern of pubertal changes in boys. Archives of Disease in Childhood, 45(239), 13–23. 10.1136/adc.45.239.13 5440182 PMC2020414

[r36] Martin, L. A., Neighbors, H. W., & Griffith, D. M. (2013). The experience of symptoms of depression in men vs women: analysis of the National Comorbidity Survey Replication. JAMA Psychiatry, 70(10), 1100–1106. 10.1001/jamapsychiatry.2013.1985 23986338

[r37] Mendle, J., Beltz, A. M., Carter, R., & Dorn, L. D. (2019). Understanding puberty and its measurement: Ideas for research in a new generation. Journal of Adolescent Research, 29(1), 82–95. 10.1111/jora.12371 30869839

[r38] Natsuaki, M. N., Biehl, M. C., & Ge, X. J. (2009). Trajectories of depressed mood from early Adolescence to young adulthood: The effects of pubertal timing and adolescent dating. Journal of Research on Adolescence, 19(1), 47–74. 10.1111/j.1532-7795.2009.00581.x

[r39] Northstone, K., Lewcock, M., Groom, A., Boyd, A., Macleod, J., Timpson, N., & Wells, N. (2019). The avon longitudinal study of Parents and Children (ALSPAC): An update on the enrolled sample of index children in 2019. Wellcome Open Research, 4, 51. 10.12688/wellcomeopenres.15132.1 31020050 PMC6464058

[r40] Patton, G. C., & Viner, R. (2007). Pubertal transitions in health. Lancet, 369(9567), 1130–1139. 10.1016/S0140-6736(07)60366-3 17398312

[r41] Petersen, A. C., Crockett, L., Richards, M., & Boxer, A. (1988). A self-report measure of pubertal status: Reliability, validity, and initial norms. Journal of Youth and Adolescence, 17(2), 117–133. 10.1007/BF01537962 24277579

[r42] Roberts, E., Joinson, C., Gunnell, D., Fraser, A., & Mars, B. (2020). Pubertal timing and self-harm: A prospective cohort analysis of males and females. Epidemiology and Psychiatric Sciences, 29. 10.1017/s2045796020000839 PMC7576520 33021194

[r43] Royston, P., & White, I. R. (2011). Multiple imputation by chained equations (MICE): Implementation in stata. Journal of Statistical Software, 45(4), 1–20. 10.18637/jss.v045.i04

[r44] Rubin, D. B. (1987). Multiple imputation for nonresponse in surveys. New York; Chichester: Wiley.

[r45] Rudolph, K. D., Troop-Gordon, W., Lambert, S. F., & Natsuaki, M. N. (2014). Long-term consequences of pubertal timing for youth depression: Identifying personal and contextual pathways of risk. Development and Psychopathology, 26(4 Pt 2), 1423–1444. 10.1017/S0954579414001126 25422971 PMC4631266

[r46] Sontag, L. M., Graber, J. A., & Clemans, K. H. (2011). The role of peer stress and pubertal timing on symptoms of psychopathology during early adolescence. Journal of Youth and Adolescence, 40(10), 1371–1382. 10.1007/s10964-010-9620-8 21170672

[r47] StataCorp. (2023). Stata statistical software: release 18. College Station, TX*:* StataCorp LLC.

[r48] Tanner, J. M. (1962). Growth at adolescence: with a general consideration of the effects of hereditary and environmental factors upon growth and maturation from birth to maturity (2d ed.). Oxford: Blackwell Scientific Publications.

[r49] Thapar, A., Collishaw, S., Pine, D. S., & Thapar, A. K. (2012). Depression in adolescence. Lancet, 379(9820), 1056–1067. 10.1016/S0140-6736(11)60871-4 22305766 PMC3488279

[r50] Turner, N., Joinson, C., Peters, T. J., Wiles, N., & Lewis, G. (2014). Validity of the short mood and feelings questionnaire in late adolescence. Psychological Assessment, 26(3), 752–762. 10.1037/a0036572 24749755

[r51] Ullsperger, J. M., & Nikolas, M. A. (2017). A meta-analytic review of the association between pubertal timing and psychopathology in adolescence: Are there sex differences in risk? Psychological Bulletin, 143(9), 903–938. 10.1037/bul0000106 28530427

[r52] World Health Organization (WHO). (1992). *The ICD-10 classification of mental and behavioural disorders: Clinical descriptions and diagnostic guidelines* (Vol. 1). World Health Organization.

