# Supplementary material for: Pubertal timing, depressive symptoms, and depression in adolescent males: a prospective cohort study
Source: Psychol Med. 2025 Feb 4;55:e3. doi: 10.1017/S0033291724003234 (PMC11968118; doi:10.1017/S0033291724003234)
Supplement: Tarif et al. supplementary material [file S0033291724003234sup001.docx]

**Supplementary Materials**

[S1. Participant flow chart 2](#_Toc181201652)

[S2. Associations between pubertal timing variables and depressive symptoms at age 14 in imputed sample N= 4,664 3](#_Toc181201653)

[S3. Associations between pubertal timing variables and depressive symptoms at age 18 in imputed sample N= 4,664. 4](#_Toc181201654)

[S4. Associations between pubertal timing variables and depression diagnosis at age 18 in imputed sample N= 4,664. 5](#_Toc181201655)

[S5. Associations between pubertal timing variables and depressive symptoms at 14 in the complete case. N = 986 6](#_Toc181201656)

[S6. Associations between pubertal timing variables and depressive symptoms at 18 in the complete case. N = 986 7](#_Toc181201657)

[S7. Associations between pubertal timing variables and depression diagnosis at 18 in the complete case. N = 986 8](#_Toc181201658)

[S8. Coding of Variables 9](#_Toc181201659)

[S9. Characteristics across different samples 15](#_Toc181201660)

[S10. Missing Data Approach 16](#_Toc181201661)

# S1. Participant flow chart

Boys only

N= 7,684

At least 1 puberty measure

N= 4,664 (**Imputed Sample**)

Complete data on all 7 puberty measures N= 3,129

Complete data on all 7 puberty measures and 3 depression measures

N= 1,248

Complete data on all puberty measures, depression measures and confounders

N= 986 (**Complete Case Sample**)

7,961 girls excluded

3,020 excluded for missing all 7 puberty measures

1,535 excluded for missing puberty data

N= 15,465

1,881 excluded for missing depression data

262 excluded for missing confounder data

# S2. Associations between pubertal timing variables and depressive symptoms at age 14 in imputed sample N= 4,664

|  | **Unadjusted** | | | **Adjusted for SES** | | | **Adjusted for SES and BMI at 9** | | |
| --- | --- | --- | --- | --- | --- | --- | --- | --- | --- |
|  | OR (95% CI) | | Omnibus  p-value | OR (95% CI) | | Omnibus  p-value | OR (95% CI) | | Omnibus  p-value |
|  | Early | Late |  | Early | Late |  | Early | Late |  |
| aPHV | 1.12  (0.79, 1.61) | 0.88  (079, 1.61) | 0.623 | 1.14  (0.79, 1.64) | 0.91  (0.61, 1.36) | 0.649 | 1.13  (0.79, 1.64) | 0.91  (0.61, 1.40) | 0.678 |
| Age peak weight velocity | 1.23  (0.87, 1.76) | 0.70  (0.46, 1.05) | 0.082 | 1.25  (0.88, 1.78) | 0.70  (0.46, 1.07) | 0.080 | 1.27  (0.86, 1.88) | 0.70  (0.46, 1.07) | 0.085 |
| Age peak BMC velocity | 1.23  (0.86, 1.76) | 1.03  (0.71, 1.49) | 0.505 | 1.26  (0.88, 1.81) | 1.05  (0.72, 1.53) | 0.421 | 1.26  (0.88, 1.79) | 1.06  (0.72, 1.55) | 0.437 |
| Age Tanner pubic hair stage 3 | 1.20  (0.80, 1.80) | 0.80  (0.51, 1.24) | 0.331 | 1.20  (0.80, 1.81) | 0.82  (0.53, 1.28) | 0.388 | 1.20  (0.79, 1.82) | 0.82  (0.53, 1.29) | 0.407 |
| Age Tanner genitalia stage 3 | 1.11  (0.75, 1.65) | 1.02  (0.69, 1.50) | 0.852 | 1.09  (0.74, 1.61) | 1.05  (0.71, 1.55) | 0.894 | 1.09  (0.74, 1.61) | 1.05  (0.71, 1.55) | 0.893 |
| Age axillary  hair | 0.89  (0.60, 1.31) | 1.08  (0.68, 1.70) | 0.756 | 0.90  (0.60, 1.33) | 1.10  (0.70, 1.74) | 0.752 | 0.89  (0.60, 1.33) | 1.11  (0.70, 1.74) | 0.734 |
| Age voice break | 1.07  (0.70, 1.64) | 1.06  (0.72, 1.57) | 0.919 | 1.09  (0.71, 1.68) | 1.08  (0.73, 1.61) | 0.871 | 1.09  (0.71, 1.67) | 1.08  (0.73, 1.61) | 0.875 |

*Note: Depressive Symptoms = SMFQ>=11*

*Reference: On-Time*

*SES variables include maternal education, social class, home ownership, financial problems and father absence.*

# S3. Associations between pubertal timing variables and depressive symptoms at age 18 in imputed sample N= 4,664.

|  | Unadjusted | | | Adjusted for SES | | | Adjusted for SES and BMI at 9 | | |
| --- | --- | --- | --- | --- | --- | --- | --- | --- | --- |
|  | OR (95% CI) | | Omnibus  p-value | OR (95% CI) | | Omnibus  p-value | OR (95% CI) | | Omnibus  p-value |
|  | Early | Late |  | Early | Late |  | Early | Late |  |
| aPHV | 1.15  (0.82, 1.62) | 0.93  (0.63, 1.38) | 0.637 | 1.19  (0.84, 1.66) | 0.95  (0.64, 1.43) | 0.578 | 1.27  (0.88, 1.83) | 0.92  (0.61, 1.39) | 0.385 |
| Age peak weight velocity | 1.05  (0.71, 1.54) | 1.02  (0.71, 1.46) | 0.969 | 1.06  (0.72, 1.56) | 1.02  (0.70, 1.47) | 0.947 | 1.23  (0.82, 1.86) | 1.02  (0.70, 1.48) | 0.594 |
| Age peak BMC velocity | 1.06  (0.79, 1.43) | 1.06  (0.74, 1.51) | 0.899 | 1.09  (0.81, 1.48) | 1.08  (0.75, 1.55) | 0.805 | 1.14  (0.83, 1.56) | 1.05  (0.74, 1.50) | 0.719 |
| Age Tanner pubic hair stage 3 | 1.04  (0.72, 1.51) | 1.27  (0.90, 1.80) | 0.414 | 1.05  (0.72, 1.53) | 1.31  (0.93, 1.87) | 0.332 | 1.08  (0.74, 1.57) | 1.29 (0.91, 1.84) | 0.376 |
| Age Tanner genitalia stage 3 | 1.17  (0.80, 1.71) | 1.05  (0.74, 1.51) | 0.680 | 1.15  (0.78, 1.68) | 1.09  (0.76, 1.57) | 0.708 | 1.14  (0.78, 1.67) | 1.10  (0.77, 1.58) | 0.721 |
| Age axillary  hair | 0.83  (0.59, 1.18) | 0.83  (0.54, 1.28) | 0.452 | 0.86  (0.60, 1.22) | 0.85  (0.55, 1.32) | 0.565 | 0.88  (0.61, 1.26) | 0.83  (0.53, 1.29) | 0.579 |
| Age voice break | 1.36  (0.94, 1.98) | 1.27  (0.88, 1.82) | 0.153 | 1.40  (0.96, 2.04) | 1.29  (0.89, 1.86) | 0.125 | 1.43  (0.98, 2.09) | 1.28  (0.88, 1.85) | 0.105 |

*Note: Depressive Symptoms = SMFQ>=11*

*Reference: On-Time*

*SES variables include maternal education, social class, home ownership, financial problems and father absence.*

# S4. Associations between pubertal timing variables and depression diagnosis at age 18 in imputed sample N= 4,664.

|  | Unadjusted | | | Adjusted for SES | | | Adjusted for SES and BMI at 9 | | |
| --- | --- | --- | --- | --- | --- | --- | --- | --- | --- |
|  | OR (95% CI) | | Omnibus  p-value | OR (95% CI) | | Omnibus  p-value | OR (95% CI) | | Omnibus  p-value |
|  | Early | Late |  | Early | Late |  | Early | Late |  |
| aPHV | 2.11  (1.35, 3.30) | 0.95  (0.46, 1.94) | 0.008 | 2.23  (1.41, 3.52) | 0.99 (0.48, 2.04) | 0.005 | 2.06  (1.27, 3.34) | 1.03  (0.50, 2.13) | 0.019 |
| Age peak weight velocity | 2.26  (1.30, 3.91) | 1.15  (0.56, 2.37) | 0.015 | 2.36  (1.35, 4.14) | 1.17  (0.56, 2.42) | 0.011 | 2.10  (1.16, 3.79) | 1.16  (0.56, 2.39) | 0.060 |
| Age peak BMC velocity | 1.46  (0.85, 2.51) | 1.03  (0.53, 2.00) | 0.375 | 1.55  (0.90, 2.66) | 1.07  (0.55, 2.08) | 0.291 | 1.40  (0.81, 2.43) | 1.14  (0.58, 2.23) | 0.480 |
| Age Tanner pubic hair stage 3 | 1.17  (0.67, 2.07) | 0.67  (0.27, 1.65) | 0.514 | 1.18  (0.67, 2.07) | 0.70  (0.28, 1.75) | 0.581 | 1.10  (0.62, 1.97) | 0.73  (0.29, 1.82) | 0.701 |
| Age Tanner genitalia stage 3 | 1.81  (1.02, 3.20) | 0.81  (0.37, 1.77) | 0.076 | 1.75  (0.97, 3.14) | 0.85  (0.38, 1.87 | 0.121 | 1.81  (1.01, 3.26) | 0.81  (0.36, 1.82) | 0.090 |
| Age axillary  hair | 1.01  (0.56, 1.83) | 0.94  (0.43, 2.06) | 0.986 | 1.05  (0.58, 1.92) | 0.97  (0.44, 2.16) | 0.982 | 1.00  (0.54, 1.84) | 1.02  (0.46, 2.28) | 0.998 |
| Age voice break | 1.89  (1.02, 3.49) | 0.98  (0.53, 1.84) | 0.072 | 1.98  (1.05, 3.72) | 1.02  (0.54, 1.92) | 0.058 | 1.90  (0.99, 3.62) | 1.03  (0.54, 1.94) | 0.088 |

*Note: Reference: On-Time*

*SES variables include maternal education, social class, home ownership, financial problems and father absence.*

# S5. Associations between pubertal timing variables and depressive symptoms at 14 in the complete case. N = 986

|  | **Unadjusted** | | | **Adjusted for SES** | | | **Adjusted for SES and BMI at 9** | | |
| --- | --- | --- | --- | --- | --- | --- | --- | --- | --- |
|  | OR (95% CI) | | Omnibus  p-value | OR (95% CI) | | Omnibus  p-value | OR (95% CI) | | Omnibus  p-value |
|  | Early | Late |  | Early | Late |  | Early | Late |  |
| aPHV | 1.13  (0.61, 2.12) | 1.33  (0.68, 2.58) | 0.697 | 1.10  (0.59, 2.08) | 1.40  (0.71, 2.74) | 0.620 | 1.09  (0.57, 2.09) | 1.41  (0.71, 2.80) | 0.613 |
| Age peak weight velocity | 0.99  (0.51, 1.91) | 0.81  (0.40, 1.64) | 0.843 | 1.00  (0.52, 1.94) | 0.76  (0.37, 1.55) | 0.740 | 1.01  (0.49, 2.05) | 0.76  (0.37, 1.55) | 0.740 |
| Age peak BMC velocity | 1.27  (0.71, 2.26) | 0.89  (0.71, 2.26) | 0.628 | 1.24  (0.69, 2.22) | 0.90  (0.44, 1.85) | 0.688 | 1.25  (0.69, 2.24) | 0.89  (0.43, 1.84) | 0.679 |
| Age Tanner pubic hair stage 3 | 1.05  (0.56, 1.99) | 0.65  (0.29, 1.47) | 0.551 | 1.05  (0.55, 2.00) | 0.66  (0.29, 1.49) | 0.569 | 1.05  (0.55, 2.01) | 0.97  (0.47, 1.97) | 0.566 |
| Age Tanner genitalia stage 3 | 1.24  (0.64, 2.41) | 0.96  (0.48, 1.96) | 0.790 | 1.18  (0.61, 2.31) | 0.97  (0.47, 1.97) | 0.869 | 1.18  (0.61, 2.31) | 1.23  (0.61, 2.48) | 0.868 |
| Age axillary  hair | 0.86  (0.47, 1.59) | 1.20  (0.60, 2.39) | 0.729 | 0.88  (0.48, 1.62) | 1.22  (0.61, 2.46) | 0.729 | 0.87  (0.47, 1.62) | 0.65  (0.29, 1.49) | 0.720 |
| Age voice break | 1.97  (1.08, 3.60) | 0.66  (0.29, 1.48) | 0.031 | 2.13  (1.16, 3.93) | 0.71  (0.31, 1.61) | 0.023 | 2.13  (1.16, 3.94) | 0.71  (0.31, 1.61) | 0.023 |

*Note: Depressive Symptoms = SMFQ>=11*

*Reference: On-Time*

*SES variables include maternal education, social class, home ownership, financial problems and father absence.*

# S6. Associations between pubertal timing variables and depressive symptoms at 18 in the complete case. N = 986

|  | **Unadjusted** | | | **Adjusted for SES** | | | **Adjusted for SES and BMI at 9** | | |
| --- | --- | --- | --- | --- | --- | --- | --- | --- | --- |
|  | OR (95% CI) | | Omnibus  p-value | OR (95% CI) | | Omnibus  p-value | OR (95% CI) | | Omnibus  p-value |
|  | Early | Late |  | Early | Late |  | Early | Late |  |
| aPHV | 1.14  (0.73, 1.77) | 1.02  (0.61, 1.70) | 0.852 | 1.26  (0.72, 1.76) | 1.07  (0.64, 1.79) | 0.864 | 1.26  (0.80, 1.99) | 0.97  (0.57, 1.63) | 0.584 |
| Age peak weight velocity | 1.04  (0.65, 1.67) | 1.00  (0.65, 1.67) | 0.983 | 1.06  (0.66, 1.69) | 0.96  (0.59, 1.55) | 0.952 | 1.32  (0.79, 2.20) | 0.98  (0.60, 1.59) | 0.546 |
| Age peak BMC velocity | 1.11  (0.72, 1.71) | 1.10  (0.69, 1.78) | 0.850 | 1.10  (0.71, 1.69) | 1.13  (0.70, 1.82) | 0.849 | 1.17  (0.75, 1.81) | 1.05  (0.65, 1.71) | 0.791 |
| Age Tanner pubic hair stage 3 | 1.15  (0.73, 1.83) | 1.06  (0.64, 1.74) | 0.824 | 1.16  (0.73, 1.84) | 1.09  (0.66, 1.80) | 0.799 | 1.20  (0.76, 1.91) | 1.04  (0.62, 1.72) | 0.737 |
| Age Tanner genitalia stage 3 | 1.03  (0.62, 1.69) | 1.02  (0.62, 1.66) | 0.993 | 1.01  (0.61, 1.67) | 1.06  (0.65, 1.74) | 0.973 | 0.98  (0.59, 1.63) | 1.07  (0.65, 1.76) | 0.957 |
| Age axillary  hair | 0.74  (0.48, 1.15) | 0.75  (0.433, 1.31) | 0.302 | 0.76  (0.49, 1.17) | 0.78  (0.45, 1.36) | 0.367 | 0.80  (0.52, 1.25) | 0.75  (0.43, 1.30) | 0.425 |
| Age voice break | 1.51  (0.93, 2.46) | 1.44  (0.91, 2.23) | 0.115 | 1.61  (0.99, 2.64) | 1.49  (0.94, 2.37) | 0.068 | 1.66  (1.01, 2.72) | 1.49  (0.94, 2.37) | 0.058 |

*Note: Depressive Symptoms = SMFQ>=11*

*Reference: On-Time*

*SES variables include maternal education, social class, home ownership, financial problems and father absence.*

# S7. Associations between pubertal timing variables and depression diagnosis at 18 in the complete case. N = 986

|  | **Unadjusted** | | | **Adjusted for SES** | | | **Adjusted for SES and BMI at 9** | | |
| --- | --- | --- | --- | --- | --- | --- | --- | --- | --- |
|  | OR (95% CI) | | Omnibus  p-value | OR (95% CI) | | Omnibus  p-value | OR (95% CI) | | Omnibus  p-value |
|  | Early | Late |  | Early | Late |  | Early | Late |  |
| aPHV | 2.92  (1.48, 5.75) | 1.66  (0.69, 4.00) | 0.008 | 2.92  (1.47, 5.79) | 1.77  (0.73, 4.29) | 0.009 | 2.48  (1.22, 5.06) | 2.03  (0.82, 5.01) | 0.029 |
| Age peak weight velocity | 2.64  (1.29, 5.43) | 1.99  (0.91, 4.34) | 0.021 | 2.76  (1.34, 5.69) | 1.95  (0.89, 4.29) | 0.018 | 2.19  (1.00, 4.78) | 1.87  (0.85, 4.13) | 0.095 |
| Age peak BMC velocity | 1.38  (0.66, 2.87) | 1.46  (0.66, 3.23) | 0.536 | 1.35  (0.64, 2.83) | 1.49  (0.68, 3.31) | 0.534 | 1.23  (0.58, 2.59) | 1.74  (0.78, 3.92) | 0.398 |
| Age Tanner pubic hair stage 3 | 1.16  (0.54, 2.48) | 0.44  (0.13, 1.46) | 0.346 | 1.18  (0.55, 2.54) | 0.46  (0.14, 1.52) | 0.368 | 1.09  (0.50, 2.36 | 0.50  (0.15, 1.66) | 0.487 |
| Age Tanner genitalia stage 3 | 1.34  (0.60, 3.00) | 0.94  (0.38, 2.30) | 0.744 | 1.30  (0.58, 2.9) | 0.96  (0.39, 2.38) | 0.804 | 1.37  (0.61, 3.08) | 0.90  (0.36, 2.24) | 0.704 |
| Age axillary  hair | 0.98  (0.48, 2.00) | 0.65  (0.22, 1.88) | 0.726 | 0.99  (0.48, 2.02) | 0.68  (0.23, 1.97) | 0.768 | 0.89  (0.43, 1.84) | 0.72  (0.25, 2.10) | 0.815 |
| Age voice break | 1.50  (0.67, 3.34) | 0.91  (0.37, 2.23) | 0.569 | 1.61  (0.71, 3.64) | 0.96  (0.39, 2.37) | 0.490 | 1.55  (0.68, 3.52) | 0.95  (0.38, 2.36) | 0.545 |

*Note: Reference: On-Time*

*SES variables include maternal education, social class, home ownership, financial problems and father absence.*

# S8. Coding of Variables

| **Variables** | **Description and source of data collection** | | **Data type** | **Coding of variable** |
| --- | --- | --- | --- | --- |
| Pubertal timing variables | | | | |
| Age at peak height velocity (aPHV) | Derived from nine repeated measures of height (to nearest 0.1cm) using stadiometer from mean age 7.6 to 17.8 by accredited fieldworkers. | | Continuous – Age (in years)  Categorical – Early, On-time, Late. (Mean +/- 1 SD) | Early: < 12.7 years  On-time: 12.7 to 14.3 years  Late: > 14.3 years |
| Age at peak weight velocity (aPWV) | Derived from nine repeated measures of weight (to nearest 0.1kg) using Tanita Body Fat Analyser from mean age 7.6 to 17.8 by accredited fieldworkers. | |  | Early: < 12.3 years  On-time: 12.3 to 14.8 years  Late: > 14.8 years |
| Age at peak BMC velocity (aPBMCV) | Derived from nine repeated measures of total body (less head) bone mineral content (BMC; in grams) using Lunar Prodigy Dual-energy X-ray Absorptiometry (DXA) scans from mean age 9.9 to 17.8 by accredited fieldworkers. | |  | Early: <13.3 years  On-time: 13.3 to 14.7 years  Late: > 14.7 years |
| Age in Tanner stage 3 of pubic hair development | Derived from nine puberty questionnaires reported by parent and/or child from mean age 8.2 to 17.0 years, using line drawings with accompanying descriptions of the five Tanner stages of pubic hair development to identify the stage most closely matched with the child’s current stage. | |  | Early: < 11.6 years  On-time: 11.6 to 13.6 years  Late: > 13.6 years |
| Age in Tanner stage 3 of genitalia development | Derived from nine puberty questionnaires reported by parent and/or child from mean age 8.2 to 17.0 years, using line drawings with accompanying descriptions of the five Tanner stages of genitalia development to identify the stage most closely matched with the child’s current stage. | |  | Early: < 11.5 years  On-time: 11.5 to 14.0 years  Late: > 14.0years |
| Age at voice break | Derived from eight puberty questionnaires reported by parent and/or child from mean age 9.7 to 17.0 years, asking whether the participant’s voice (1) had not changed, (2) was occasionally a lot lower, or (3) had changed totally. | |  | Early: < 13.1 years  On-time: 13.1 to 15.4 years  Late: > 15.4 years |
| Age at axillary hair | Derived from seven puberty questionnaires by parent and/or child from mean age 9.7 to 17.0 years, asking whether the participant had (1) started growing hair in the armpits or (2) not yet started. | |  | Early: < 12.4 years  On-time 12.4 to 14.4 years  Late: > 14.4 years |
| Depression Variables | | | | |
| Depressive Symptoms (SMFQ) | Var name: FG7226 at 13.84 years and CCXD917 at 17.84 years  Short Moods and Feeling Questionnaire (SMFQ) | | Continuous variable ranging from 0-26  Categorical Variable: Cut-off point at 11  0: No depressive symptoms; 1: Depressive Symptoms | (<11=0), (≥11=1)  0: No  1: Yes  -10, -1: Missing |
| Depression Diagnosis at 18 (CIS-R) | Var name: FJCI1001  ICD-10 Diagnosis of depression (any severity: mild, moderate or severe) | | Categorical Variable:  0: Not diagnosed  1: Diagnosed | 0: No  1: Yes  -10, -4, -1: Missing |
| Confounding Variables | | | | |
| Home ownership | Mother completed; sent when child was 1y9m  Var name: g352 | | Categorical Var  0 - House rented from council/housing association/bought from council  1 - House privately rented/ owned/mortgaged | Recode (0 2 4 5 = 1) (1 3 6 =0) (-1 7 = .) |
|  | Is your home: |  |  |  |
|  | Being bought/mortgaged | 0 |  |  |
|  | Being bought from council | 1 |  |  |
|  | Owned – with no mortgage | 2 |  |  |
|  | Rented from council | 3 |  |  |
|  | Rented from private landlord (furnished) | 4 |  |  |
|  | Rented from private landlord (unfurnished) | 5 |  |  |
|  | Rented from housing association | 6 |  |  |
|  | Other | 7 |  |  |
|  | Missing | -1 |  |  |
| Maternal Education | Mother completed; sent when child was 5y1m  1 Yes  -1 No Response | | Categorical Variable  0 = < O Level  1 = O Level  2 = > O Level | 0 if k6280==1 \| k6281==1 \| k6284==1 \| k6285==1 \|k6286==1 \| k6288==1 \| k6289==1 \| k6290==1  1 if k6282==1  2 if k6283==1 \| k6291==1 \| k6292==1 \| k6287==1 |
|  | k6280 | Mother has/is:  No educational quals |  |  |
|  | k6281 | CSE/GCSE |  |  |
|  | k6284 | Vocational qual |  |  |
|  | k6285 | Apprenticeship |  |  |
|  | k6286 | State enrolled nurse |  |  |
|  | k6288 | City & Guilds Intermediate Technical Quals |  |  |
|  | k6289 | City & Guilds Final Technical Quals |  |  |
|  | k6290 | City & Guilds Full Technical Quals |  |  |
|  | k6282 | O-Level/GCSE |  |  |
|  | k6283 | A Levels |  |  |
|  | k6291 | Teaching Qual |  |  |
|  | k6292 | University Degree |  |  |
|  | k6287 | State Registered Nurse |  |  |
| Major Financial problems | Mother completed; sent when child was <1, 2 and 5 years old. | | Categorical Variable  0 - No financial Problems  1 - Financial Problems | 0 if f244a==2 \| h234a==2 \| k4024==5  1 if f244a==1 \| h234a==1 \| k4024==1 \| k4024==2 \| k4024==3 \| k4024==4 |
|  | f244a | “You had major financial problems”  1 Yes  2 No  -1 Missing |  |  |
|  | h234a |  |  |  |
|  | k4024 | Mother had major financial problems in past year  1 Yes, affected a lot  2 Yes, moderately  3 Yes, Mildly  4 Yes, did not affect  5 No, did not happen  -1 No Response |  |  |
| Social Class | Mother completed; sent prior to birth  C755 – maternal social class  C765 – partner social class  1 I  2 II  3 III (Non-Manual)  4 III (Manual)  5 IV  6 V  65 Armed Forces  -1 Missing | | Categorical  0 Non-Manual  1 Manual | 0 if response is 1 2 3 4  1 if response is 5 6  Missing if -1 65 |
| Father absence | Recorded by mother at various timepoints  “How old was the child when the natural father stopped living with the child?” | | Categorical  0 Father present  1 Father left before 5 years  2 Father left between 5-10 years |  |
| BMI at 9 | Calculated at age 9 based on height and weight measurements obtained from clinics and questionnaire data when clinic data was missing.  F9ms026a, pub205 | | Continuous Measure |  |
| Auxiliary Variables | | | | |
| BMI at 7 | Calculated at age 9 based on height and weight measurements obtained from clinics and questionnaire data when clinic data was missing.  f7ms026a, f7003c | | Continuous Variable |  |
| Crowding Index | Measured at 8 weeks gestation  A551  Derived: Number of people in the household (a550) divided by number of rooms (a045) | | Categorical  1 <=0.5  2 >0.5 – 0.75  3 >0.75 -1  4 > 1  -7 / -1 Missing |  |
| Car Access | Measured at 8 weeks gestation  A053  “Do you or your partner have the use of a car (including vans, minibuses, etc.)?  1 Yes  2 No  -7 / -1 Missing | | Categorical Variable  1 Yes  0 No | Recode (2=0) |
| Weekly Income | Measured when child was 47 months  J410  “On average, how much is the take home family income each week (include social benefits etc.)?”  1 < £100  2 £100 - £199  3 £200 - £299  4 £300- £399  5 > £400  -1 Missing | | Categorical Variable  1 < £100  2 £100 - £199  3 £200 - > £400  4 £300- £399  5 > £400 |  |

| Depressive Symptoms at age 13 | Short Moods and Feeling Questionnaire (SMFQ) Measured at 13 years  Derived var from FF6500-FF6515 | Continuous variable ranging from 0-26 |  |
| --- | --- | --- | --- |
| Strengths and Difficulties at age 12 | Strength and Difficulties  Derived Variable: Continuous score (Range 0-40)  kw6602a – Emotional symptoms score | Categorical variable | Recode (4/10 = 4) |
| DAWBA age 16 | DAWBA (Development and Wellbeing Assessment) Band Prediction for Depression. Measured at 16 years.  fh6876  The categories range from <0.1% (0.1% children in this band have depression) to > 70% (>70% of children in this band have depression) | Categorical  1: <0.1%  2: ~0.5%  3 >15% | Recode (5 4=3) |
| Maternal depression | Edinburgh Postnatal depression scale | Continuous Variable |  |

# S9. Characteristics across different samples

|  | Whole sample  *Up to N= 7,684* | Missing puberty data  *Up to N= 3,020* | Imputed Sample  (Not missing puberty data)  *Up to N=4,664* | Complete Case  *N= 986* | % Missing  *(In imputation sample)* |
| --- | --- | --- | --- | --- | --- |
| Mean (SE) |  |  |  |  |  |
| Age peak height velocity (in years) | 13.5 (0.8) | - | 13.5 (0.8) | 13.4 (0.9) | 0% |
| Age peak weight velocity (in years) | 13.6 (1.2) | - | 13.6 (1.2) | 13.5 (1.4) | 0% |
| Age peak BMC velocity (in years) | 14.0 (0.7) | - | 14.0 (0.7) | 13.9 (0.8) | 0% |
| Age Tanner pubic hair stage 3 (in years) | 12.6 (1.0) | - | 12.6 (1.0) | 12.6 (1.0) | 0% |
| Age Tanner genitalia stage 3 (in years) | 12.7 (1.2) | - | 12.7 (1.2) | 12.8 (1.3) | 0% |
| Age voice break (in years) | 14.2 (1.1) | - | 14.2 (1.1) | 14.3 (1.2) | 0% |
| Age axillary hair (in years) | 13.4 (1.0) | - | 13.4 (1.0) | 13.5 (1.1) | 0% |
| BMI at 9 | 17.5 (2.8) | 17.7 (3.0) | 17.5 (2.7) | 17.4 (2.6) | 30.4% |
| % (SE) |  |  |  |  |  |
| Depressive symptoms at 14^†^ | 7.4 (0.5) | 5.2 (1.1) | 7.8 (0.5) | 7.0 (0.8) | 45.0% |
| Depressive symptoms at 18^†^ | 16.7 (0.9) | 22.0 (2.6) | 15.9 (0.9) | 15.0 (1.1) | 64.7% |
| Depression diagnosis at 18 | 4.5 (0.5) | 5.0 (1.3) | 4.4 (0.5) | 4.4 (0.7) | 63.4% |
| Home ownership (renting/non-homeowner) | 15.6 (0.5) | 31.7 (1.4) | 11.1 (0.5) | 4.5 (0.7) | 12.5% |
| Maternal education |  |  |  |  |  |
| < O-levels (CSE/Vocational/None) | 21.7 (0.6) | 32.7 (1.8) | 19.6 (0.7) | 11.6 (1.0) | 17.7% |
| O-Levels | 40.9 (0.7) | 39.0 (1.8) | 41.2 (0.9) | 36.5 (1.5) |  |
| Major Financial Problems | 26.4 (0.6) | 29.1 (1.1) | 25.4 (0.6) | 22.8 (1.3) | 2.9% |
| Social Class (Manual) | 19.4 (0.5) | 28.5 (1.1) | 16.2 (0.6) | 8.2 (0.9) | 5.0% |
| Father absence |  |  |  |  |  |
| < 5 years | 7.7 (0.4) | 5.4 (0.4) | 8.2 (0.4) | 5.5 (0.7) | 17.9% |
| Between 5-10 years | 20.7 (0.6) | 47.9 (1.7) | 14.3 (0.6) | 8.2 (0.9) |  |

# S10. Missing Data Approach

We compared individuals who have pubertal timing data to those with missing pubertal timing data (S9). Those with missing pubertal timing data were more likely to have depressive symptoms at age 18, major financial problems, a lower parental social class, parents who are renter/non-homeowners, a mother with lower education and father absence between 5-10 years. This suggested the data is not MCAR (missing completely at random). Our analysis included an imputed dataset on those with complete pubertal timing data. We used Multiple Imputation by Chained Equations (MICE) (Royston & White, 2011) in Stata to impute missing data. This approach relies on the assumption that data are Missing at Random (MAR), meaning any differences between missing and observed values can be accounted for by other observed values. 50 datasets were imputed (25 iterations), with parameter estimated pooled according to Rubin’s rules (Rubin, 1987). Separate imputations were conducted for each of the seven pubertal timing measures, while still including the other six measures as continuous auxiliary variables in each model. The imputation model included all variables in the analysis and relevant auxiliary variables (Table S8). These auxiliary variables included measures of socioeconomic status, maternal mental health indicators and earlier mental health indicators (such as previous assessments of depressive symptoms). While the MAR assumption is not directly testable, the inclusion of extensive auxiliary information strengthens the plausibility of this assumption, helping to minimise potential biases associated with missing data. Table S9 shows the percentage of missing in the imputation sample. Multiple imputation has been shown to eliminate bias regardless of the proportion of missing data (Madley-Dowd et al., 2019) .

**References**

Madley-Dowd, P., Hughes, R., Tilling, K., & Heron, J. (2019). The proportion of missing data should not be used to guide decisions on multiple imputation. *J Clin Epidemiol*, *110*, 63-73. <https://doi.org/10.1016/j.jclinepi.2019.02.016>

Royston, P., & White, I. R. (2011). Multiple Imputation by Chained Equations (MICE): Implementation in Stata. *Journal of Statistical Software*, *45*(4), 1 - 20. <https://doi.org/10.18637/jss.v045.i04>

Rubin, D. B. (1987). *Multiple imputation for nonresponse in surveys*. Wiley.
